# An integrated UAV growth monitoring model of Cinnamomum camphora based on whale optimization algorithm

**DOI:** 10.1371/journal.pone.0299362

**Published:** 2024-06-21

**Authors:** Rongxin Gong, Haina Zhang, Xianghui Lu, Haolong Wan, Yue Zhang, Xin Luo, Jie Zhang, Rongxiu Xie

**Affiliations:** 1 Jiangxi Provincial Engineering Research Center of Seed-Breeding and Utilization of Camphor Trees, Nanchang Institute of Technology, Nanchang, China; 2 Jiangxi Provincial Technology Innovation Center for Ecological Water Engineering in Poyang Lake Basin, Nanchang, China; National Institute of Technology Srinagar, INDIA

## Abstract

To explore an effective analysis model and method for estimating *Cinnamomum camphora*’s (*C*. *camphora*’s) growth using unmanned aerial vehicle (UAV) multispectral technology, we obtained *C*. *camphora*’s canopy spectral reflectance using a UAV-mounted multispectral camera and simultaneously measured four single-growth indicators: Soil and Plant Analyzer Development (SPAD)value, aboveground biomass (AGB), plant height (PH), and leaf area index (LAI). The coefficient of variation and equal weighting methods were used to construct the comprehensive growth monitoring indicators (CGMI) for *C*. *camphora*. A multispectral inversion model of integrated *C*. *camphora* growth was established using the multiple linear regression (MLR), partial least squares (PLS), support vector regression (SVR), random forest (RF), radial basis function neural network (RBFNN), back propagation neural network (BPNN), and whale optimization algorithm (WOA)-optimized BPNN models. The optimal model was selected based on the coefficient of determination (R^2^), normalized root mean square error (NRMSE) and mean absolute percentage error (MAPE). Our findings indicate that apparent differences in the accuracy with different model, and the WOA–BPNN model is the best model to invert the growth potential of *C*. *camphora*, the *R*^*2*^ of the model test set was 0.9020, the RMSE was 0.0722, and the MAPE was 7%. The *R*^*2*^ of the WOA–BPNN model improved by 18%, the NRMSE decreased by 33%, and the MAPE decreased by 9% compared with the BPNN model. This study provides technical support for the modern field management of *C*. *camphora* essential oil and other dwarf forestry industries.

## 1. Introduction

Crop growth refers to the state and trend of crops at different growth stages. Common crop growth parameters include PH leaf area index LAI, chlorophyll content, water, and AGB [1, 2]. Crop growth is directly related to crop yield and quality, and it must be observed and assessed to effectively support agricultural production [3, 4]. However, the traditional crop growth monitoring methods are based on visual observation or destructive sampling, which is labor-, time-, and resource-consuming and particularly inefficient when monitoring large crop area [5].

Remote sensing technology provides information rapidly and non-destructively on a large scale and has broad applications for crop growth prediction [6, 7]. Liu et al. used the DJI Phantom 4 multispectral UAV to obtain the spectral reflectance of a rice canopy and established regression models of three growth indicators and vegetation indices, including rice SPAD, AGB, and leaf area, discovering that the accuracy of the multi-vegetation index regression model was superior to that of the one-dimensional regression model and that the multi-vegetation index regression model and rice SPAD established using support vector machine had the highest accuracy [6]. However, a single-growth index characterizes the growth condition of a specific crop feature and fails to represent the crop’s comprehensive growth to some extent; therefore, several researchers have studied integrating the single-growth index. For example, Y. Xu et al. obtained AGB, PH, SPAD, and plant water content data of winter wheat and constructed the CGMI based on the coefficient of variation (CV) method; they discovered that the inversion model of CGMI and vegetation index of wheat constructed by genetic algorithm optimized neural network have the highest accuracy, with the coefficient of determination *R*^*2*^ reaching 0.8 [8]. X. Xu et al. used an improved fuzzy comprehensive evaluation (FCE) method to establish a comprehensive yield evaluation index (CYEI) to obtain information, such as wheat LAI, biomass, water content, and nitrogen content, to monitor wheat growth and estimate yield [9].

The above studies have shown that machine learning (ML) effectively builds single or comprehensive crop growth prediction models using UAV multispectral techniques. The ML models’ accuracy and efficiency depend heavily on their internal parameters, which can be improved using optimization algorithms to determine model parameters [10]. For example, Han et al. used the XGBoost model optimized by the grasshopper optimization algorithm (GOA) to establish the inversion model of multispectral vegetation index and wheat raw AGB, discovering that the GOA-XGB model (having 37 input factors) had the highest accuracy, with *R*^*2*^ = 0.847 [11]. Jianqiang et al used UAV multispectral remote sensing to establish a model for monitoring SPAD values of jujube leaves found that the PSO-ELM model using the particle swarm optimization (PSO) algorithm was more accurate than the ELM model alone, with a 7% improvement in R^2^ and a 52% decrease in RMSE [12]. Mirjalili et al. proposed the novel whale optimization algorithm (WOA), which uses random or optimal search agents to simulate hunting behavior and spirals to simulate the bubble-net attacking mechanism of humpback whales [13]. This algorithm is widely used in economic scheduling, optimal control, photovoltaic systems, image segmentation, and other fields owing to its simple mechanism, fewer parameters, high optimization capacity, and ability to escape local optima [14]. For example, the WOA algorithm can solve deep neural network architecture training and hyperparameter optimization problems [15]. In the field of remote sensing for agriculture and forestry, Feng et al. used unmanned aerial spectral remote sensing to establish a model for monitoring nitrogen content in rice, and found that the rice nitrogen content inversion model constructed by Whale-optimized Extreme Learning Machine (WOA-ELM) was superior to the rice nitrogen content inversion model constructed by Whale-optimized Extreme Learning Machine (ELM) [16]. This suggests that the WOA algorithm has great potential in the optimization of agroforestry remote sensing models. *Cinnamomum camphora* (Linn.) Presl is an ornamental tree and essential woody oil plant in China. Because of the oil’s anti-inflammatory, anti-bacterial, and pain-relieving properties, it is also exported to other countries as a spice and holds significance in the medical and spice industry owing to its high economic value [17–19]. *C*. *camphora* essential oil industry is developing rapidly and has emerged as a leading forestry industry in southern China [20]. *C*. *camphora* growth directly affects its oil yield and essential oil quality. Therefore, *C*. *camphora* growth must be rapidly and accurately monitored, and the development of field management systems for forestry production must be timely directed to develop the high-quality *C*. *camphora* essential oil industry. However, the research on *C*. *camphora* growth monitoring only stays on the inversion of a single growth index, and some researchers have established the inversion model of spectral index on camphor SPAD and oil yield using UAV multispectral remote sensing images, and found that radial basis function neural network and random forest are the best models for inversion of spectral index on camphor SPAD and oil yield, with the coefficients of decision of 0.79 [21] and 0.87 [22], respectively, and there are few studies on the use of multiple growth indicators to realize comprehensive monitoring of *C*. *camphora* growth.

In this study, we obtained *C*. *camphora*’s canopy spectral reflectance using a UVA-mounted multispectral camera and simultaneously measured four single-growth indicators: SPAD, AGB, PH, and LAI. The CV and equal weighting methods were used to construct *C*. *camphora* comprehensive growth monitoring indicators. The multiple linear regression (MLR), partial least squares regression (PLS), support vector regression (SVR), random forest (RF), radial basis function neural network (RBFNN), back propagation neural networks (BPNN), and WOA–BPNN models were used to establish an efficient multispectral inversion model for the comprehensive growth of *C*. *camphora* and an efficient method for estimating *C*. *camphora* growth, providing technical support for modern field management of *C*. *camphora* essential oil and other dwarf forestry industries.

## 2. Materials and methods

### 2.1 Overview of the study area

The study site was located in the *C*. *camphora* Germplasm Resource Nursery, Nanchang Institute of Technology, Nanchang, Jiangxi Province, (28°41′40.85″N, 116°1′41.18″E). The study area had a subtropical humid seasonal-wind climate with sufficient light and an average annual rainfall of 1600 mm, with precipitation of 147–157 d and an average multi-year temperature of approximately 17°C. The average annual sunshine hours were approximately 1772–1845 h, with a sunshine rate of 40%. Some of the soils in the area are dense and have a reddish soil texture that is weakly acidic. The soil included: organic matter content, 6.39 g·kg^-1^; total nitrogen content, 0.62 g·kg^-1^; total phosphorus content 0.30 g·kg^-1^; total potassium content, 13.00 g·kg^-1^; alkaline hydrolysis nitrogen content, 47.74 mg·kg^-1^; fast-acting phosphorus content, 1.49 mg·kg^-1^; fast-acting potassium content, 61.10 mg·kg^-1^.

**Fig 1:**
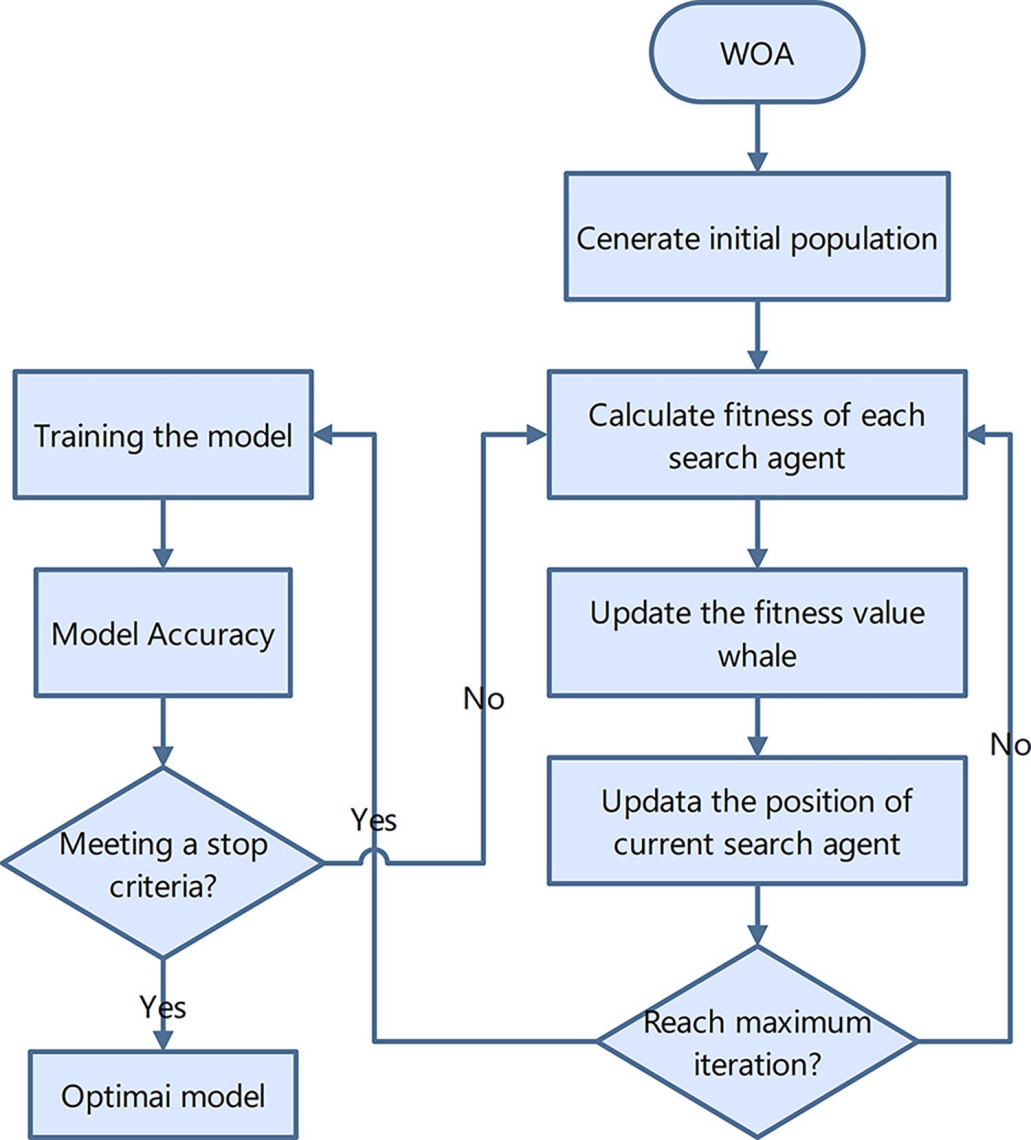
Flowchart of whale flock optimization algorithm.

The test *C*. *camphora* species was “Ganfang No. 1”. The study area was divided into 66 plots (Fig 1), each planted with nine *C*. *camphora* seedlings in three rows and three columns with a plant spacing of 1 m. Each sample plot was 3 m × 3 m, with 594 one-year-old cutting seedlings planted in April 2021.

### 2.2 Data acquisition

#### 2.2.1 Multispectral data acquisition

An MS600pro multispectral camera platform (Yusense, Inc., Qingdao, China), manufactured by Changguang Yuchen Information Technology and Equipment (Qingdao) Co., Ltd., mounted on DJI Matrice M300RTK Quadrotor UAV was used to acquire remote sensing data. The multispectral camera had six spectral channels. The multispectral image was taken on the morning of September 25, 2022, in clear and cloudless weather. The flight route was planned based on the study area, and whiteboard correction was performed. The following conditions were applied: flight altitude, 30 m; setting speed, 2.5 m·s^-1^; resolution of the image element, 4.09 cm; automatic capture mode, with a 75% heading overlap and 65% side overlap.

The obtained UAV images were spliced and preprocessed with geometric and radiometric corrections using Yusense Map aerial remote sensing preprocessing software. Subsequently, the preprocessed UAV multispectral image information was imported into ENVI software, each plot in the study area was considered a region of interest (ROI), and *C*. *camphora* leaf images were intercepted to remove the impact bands of soil and shadows (Fig 1). The average reflectance spectrum of *C*. *camphora* leaf samples in each ROI was used as the plot spectral reflectance.

#### 2.2.2 *C*. *camphora* growth index collection

The *C*. *camphora* growth index was simultaneously obtained with the UAV flight. Three good-growing *C*. *camphora* trees were selected in each plot, and five healthy mature leaves from the upper and middle parts of each tree were randomly selected to measure their SPAD using a SPAD-502 handheld chlorophyll meter. Each leaf part was measured thrice to obtain an average value, and the average value of five leaf measurements was calculated as the tree SPAD. A tape was used to measure the height of nine *C*. *camphora* plants in each plot, which was the distance of the plant from the ground to the highest point of the branches, and the average was calculated as the PH of the plot. The LAI data were measured using the LAI-2200C plant canopy analyzer (USA). Three representative *C*. *camphora* trees were selected for each sample plot, and LAI values were measured and recorded at four selected sites for each tree; the average of the four measurements was calculated as the LAI of the tree, and the average of the three trees was calculated as the LAI of the plot. After bringing three representative *C*. *camphora* trees from each sample plot to the laboratory, they were first heated at 105°C for 30 min, then baked at 80°C until constant mass, and weighed using an electronic balance. The mean value was calculated for each plot to obtain the AGB.

### 2.3 Vegetation index construction

Vegetation indices are quantitative values derived from multispectral remote sensing data about the Earth’s vegetation cover status, typically obtained through mathematical operations using the red and near-infrared bands. Table 1 shows the calculation formulae for various common vegetation indices, where B, G, R, RE, and NIR are the spectral reflectance of 450, 550, 660, 750, and 840 nm bands, respectively.

**Table 1:** Vegetation index calculation formula.

Vegetation Index	Formula	Literature
Normalized Differential Vegetation Index (NDVI)	(NIR−R)/(NIR+R)	[23]
Greenness Normalized Differential Vegetation Index (GNDVI)	(NIR−G)/(NIR+G)	[23]
Modified Simple Ratio (MSR)	($(NIR/R)-1)/\sqrt{(NIR/R)+1}$	[24]
Transformed Chlorophyll Absorption Reflectance Index (TCARI)	3×(RE−R)−0.2×(RE−G)×(RE/R)	[25]
Red-edge Chlorophyll Index (CIrededge)	*NIR*/(*RE*−1)	[26]
Difference Vegetation Index (DVI)	*NIR*−*R*	[27]
Optimized Soil Adjusted Vegetation Index (OSVAVI)	(NIR−R)/(NIR+R+0.16)	[28]
Visible Difference Vegetation Index (VDVI)	(2G−R−B)/(2G+R+B)	[29]
Structural Insensitivity Index (SIPI)	(NIR−B)/(NIR+B)	[30]
Modified Triangular Vegetation Index (MTVI)	$\frac{1.5(1.2(NIR-G)-2.5(R-G)}{\sqrt{(2NIR+1{)}^{2}-0.5-(NIR-5\sqrt{R}})}$	[31]
Modified Nonlinear Vegetation Index (MNVI)	$1.5({NIR}^{2}-2)/({NIR}^{2}+R+0.5)$	[32]
Atmospheric Resistance Vegetation Index (ARVI)	(NIR−(2R−B)/(NIR+(2R−B))	[33]

### 2.4 Comprehensive growth index construction

To better understand the growth status of *C*. *camphora* in the study area, four vegetation growth indicators: SPAD, PH, LAI, and AGB, were selected to construct a comprehensive growth monitoring index (CGMI). The four single-growth indicators were normalized to eliminate the dimension and were used to construct CGMI_1_ with equal weights using the following formula [34]:

$$U_{i}=\frac{x_{i}-{minx}_{i}}{{maxx}_{i}-{minx}_{i}}$$ (1)

$${CGMI}_{1}=\frac{1}{4}\sum_{i=1}^{4}U_{i}$$ (2)

where *i* is the index category, *U*_*i*_ is the normalized index of *i*, and *maxx*_*i*_ and *minx*_*i*_ are the maximum and minimum values of the index of *i* category, respectively.

The CV method is a common weight determination method that uses the degree of variation of each evaluation index to determine its weight; the given index weight increases or decreases with increasing or decreasing CV, respectively [35]. Because the CV reflects the relative fluctuation of indicators, indicators with large coefficients of variation reflect more important information and have higher differentiation and stability, which are given larger weights; conversely, the evaluation indicators are given smaller weights. The CV, i.e., the standard deviation of the indicator divided by the mean, is calculated as [36]:

$$S_{i}=\sqrt{\frac{1}{4}\sum_{i=1}^{4}(U_{i-{\bar{x}}_{i}}}{)}^{2}$$ (3)

$$V_{i}=\frac{S_{i}}{{\bar{x}}_{i}}$$ (4)

where ${\bar{x}}_{i}$ is the average of index *i*, *S*_*i*_ is the standard deviation of index *i*, and *V*_*i*_ is the CV of index *i*.

The CV method was used to determine the weights of the normalized SPAD, PH, LAI, and AGB vegetation growth indicators and construct CGMI_2_ using the formula [34]:

$$W_{i}=\frac{V_{i}}{\sum_{i=1}^{4}V_{i}}$$ (5)

$${CGMI}_{2}=\sum_{i=1}^{4}U_{i}W_{i}$$ (6)

where *W*_*i*_ is the index weight of *i*.

### 2.5 Model construction

#### 2.5.1 Multiple linear regression (MLR)

MLR is a widely used statistical model for predicting a continuous dependent variable from multiple independent variables. Unlike simple linear regression, which utilizes only one single independent variable, MLR can accommodate multiple independent variables, increasing the complexity and accuracy of the prediction model. It is important to note that the MLR model performs better for cases where there is no high degree of covariance (i.e., there is no linear correlation between the independent variables) between the independent variables. When there is a high degree of covariance between the independent variables, the model results may be misleading. In practice, regression diagnostics and model validation are usually required to assess the applicability and accuracy of MLR models. [37]. The MLR model for this study was built using Matlab software, setting Modelspec as linear.

#### 2.5.2 Support vector regression (SVR)

Support Vector Machines (SVMs) were originally proposed for binary classification problems. It is a powerful supervised learning algorithm designed to find an optimal hyperplane for classification in a high-dimensional feature space. SVR uses the same principles as SVM, but focuses on modeling and predicting continuous variables rather than category labels. It is particularly well suited for solving nonlinear regression problems. In SVR, a kernel function (e.g. radial basis function RBF or polynomial kernel function) is used to transform the feature space into a high-dimensional space. By projecting the data into this high-dimensional space, SVR can capture more complex relationships between features and target variables. The choice of kernel function plays a key role in determining the mapping from the original feature space to the high-dimensional space. Overall, SVR is an important extension of Support Vector Machines that applies a kernel function to transform the feature space into a nonlinear regression model.[38, 39]. In this study, a Gaussian RBF was used as the kernel function, and a network search method was used to select optimal parameters, with an RBF parameter of 30 and a penalty factor of 20.

#### 2.5.3 Random Forest (RF)

Random Forest (RF) is an integrated learning method that consists of multiple decision trees, and its basic principle is to improve prediction accuracy and robustness through the integration of multiple decision trees. Each decision tree uses randomly selected features and samples during training, so that each decision tree learns different features and laws from different subsets of data. When a prediction is needed, each decision tree makes an independent prediction and finally the final prediction is determined by voting. Overall, Random Forest is a powerful integrated learning method that can effectively deal with classification and regression problems. It improves the prediction performance and generalization ability through the mechanism of decision tree integration and random attribute selection, with good robustness and interpretability.[40]. A 10-fold cross-validated grid search method was used to adjust RF parameters, setting the number of leaf nodes to 4 and the number of decision trees to 100.

#### 2.5.4 Partial least squares (PLS)

Partial Least Squares Regression (PLS) combines the advantages of typical correlation analysis, principal component analysis, and multiple linear regression to deal with high-dimensional data and multiple covariates, and to solve multivariate problems. The core idea of PLS is to find the maximum covariance between the independent and dependent variables by projecting them in the Direction. [41]. The PLS model for this study was built using Matlab software, setting NCOMP as 2.2.5.5 Radial basis function neural network (RBFNN)

Radial Basis Function Neural Network (RBFNN) is a nonparametric regression method that uses radial basis functions as basis functions to fit continuous nonlinear functions. The core idea of RBFNN is to use radial basis functions as activation functions in the hidden layer of the network. These radial basis functions are basis functions parameterized by the distance between the input variables and their respective centers. The neurons in the hidden layer are activated using these radial basis functions and their outputs are linearly combined with the input variables as weights. Finally, the output layer is linearly combined to obtain the final prediction.[42, 43]. RBFNN has only one linear layer in its hidden layer, which reduces the number of iterations and computation time and improves the training speed compared with traditional feedforward neural networks. The expansion speed of RBF was set to 100.

#### 2.5.5 Back propagation neural network (BPNN)

BPNN is an artificial neural network based on an error backpropagation algorithm. Its basic structure comprises an input layer, a hidden layer, and an output layer. BPNN can build a nonlinear mapping relationship by combining multiple layers of neurons. By changing the weights and bias of the hidden layer, BPNN can approximate the nonlinear relationship between the input and output [44]. Its most prominent feature lies in the signal’s forward propagation and the error’s backward propagation, and this propagation mechanism shifts the output data closer to the expected value. BPNN can be improved by increasing intermediate layers and the number of neurons to fit different application areas and task types [45, 46]. The BPNN estimation model was constructed using MATLAB’s Neural Network toolbox. Based on the numerical optimization theory, the transfer function of the implicit layer was set to the Tansig function, and the Levenberg–Marquardt (L–M) algorithm was used for network training. After several training sessions, the number of intermediate layer neurons was set to 12, the maximum number of iterations to 1000, the training target to *e*×10^−4^, and the learning rate to 0.1. The size of the batch data used for gradient descent each time was 9, and the simulated values were obtained after the neural network training.

For a better presentation of the model, the model parameters are shown in the following Table 2.

**Table 2:** Model parameters.

Models	Tuning parameter models
MLR	MODELSPEC = ’linear’
PLS	NCOMP = 2
SVR	penalty factor: 20 RBF parameter: 30
RF	leaf nodes: 4 decision trees: 100
RBFNN	expansion speed: 100
BPNN	intermediate layer neurons: 12 maximum number of iterations: 1000 training target: e×10 ^(-4)^

### 2.6 Whale optimization algorithm (WOA)

Humpback whales have a special hunting method called bubble-net attacking method. The WOA simulates the humpback whale’s unique hunting method and seizure mechanism, which is mainly divided into searching and encircling the prey, bubble-net foraging, and obtaining the global optimal solution. First, the searching and encircling prey are simulated as follows [13]:

$$B=|2rx_{best}^{t}-x_{i}^{t}|$$ (7)

$$x_{i}^{t+1}=x_{best}^{t}-AB$$ (8)

A=2ar−a (9)

$$a=2-\frac{2t}{T}$$ (10)

where A is the coefficient vector, B is the encircling step, $x_{best}^{t}$ is the position of the individual whale with the global optimal solution within the population when iterated to generation t, $x_{i}^{t}$ is the position of the individual when iterated to generation t, r is a random number within [0,1], a is a linear decreasing coefficient from 2 to 0, and T is the maximum number of iterations.

The bubble-net attacking is simulated as follows:

$$C=|x_{best}^{t}-x_{i}^{t}|$$ (11)

$$x_{i}^{t+1}=x_{best}^{t}+Ce^{bl}\;\cos\left(2\pi l\right)$$ (12)

where C denotes the distance between the whale and the prey, b is a constant controlling the logarithmic spiral shape, and l is a random number within [–1,1]. The flowchart of the whale swarm optimization algorithm is shown in Fig 1.

### 2.7 Model accuracy evaluation

To determine the most ideal model and test its reliability and accuracy of prediction results, *R*^*2*^ 、 normalized root mean square error (NRMSE) and Mean Absolute Percentage Error (MAPE) were calculated using the following formulae [47]:

$$R^{2}=\frac{\sum_{\dot{i}=1}^{n}{\left({\hat{a}}_{i}-\bar{b}\right)}^{2}}{\sum_{i=1}^{n}{\left(a_{i}-\bar{b}\right)}^{2}}$$ (13)

$$RMSE=\sqrt{\frac{\sum_{i=1}^{n}{\left({\hat{a}}_{i}-a_{i}\right)}^{2}}{n}}$$ (14)

$$NRMSE=\frac{RMSE}{{\hat{a}}_{imax}-{\hat{a}}_{imin}}$$ (15)

$$MAPE=\frac{100\%}{n}\sum_{i}^{n}\left|\frac{{\hat{a}}_{i}-a_{i}}{a_{i}}\right|$$ (16)

where n is the number of samples, ${\hat{a}}_{i}$ is the estimated value of *C*. *camphora* growth, ${\hat{a}}_{imax}$ and ${\hat{a}}_{imin}$ are the maximum and minimum values of the estimated *C*. *camphora* growth, respectively, *a*_*i*_ is the measured value of the photosynthetic parameter of *C*. *camphora*, and $\bar{b}$ is the mean value of actual measurements of photosynthetic parameters. The model is considered accurate when *R*^*2*^ is close to 1, NRMSE and MAPE are close to 0, and prediction results are close to the measured results. In this study, the accuracy parameters of the models were statistically compared to identify the optimal model more intuitively.

**Fig 2:**
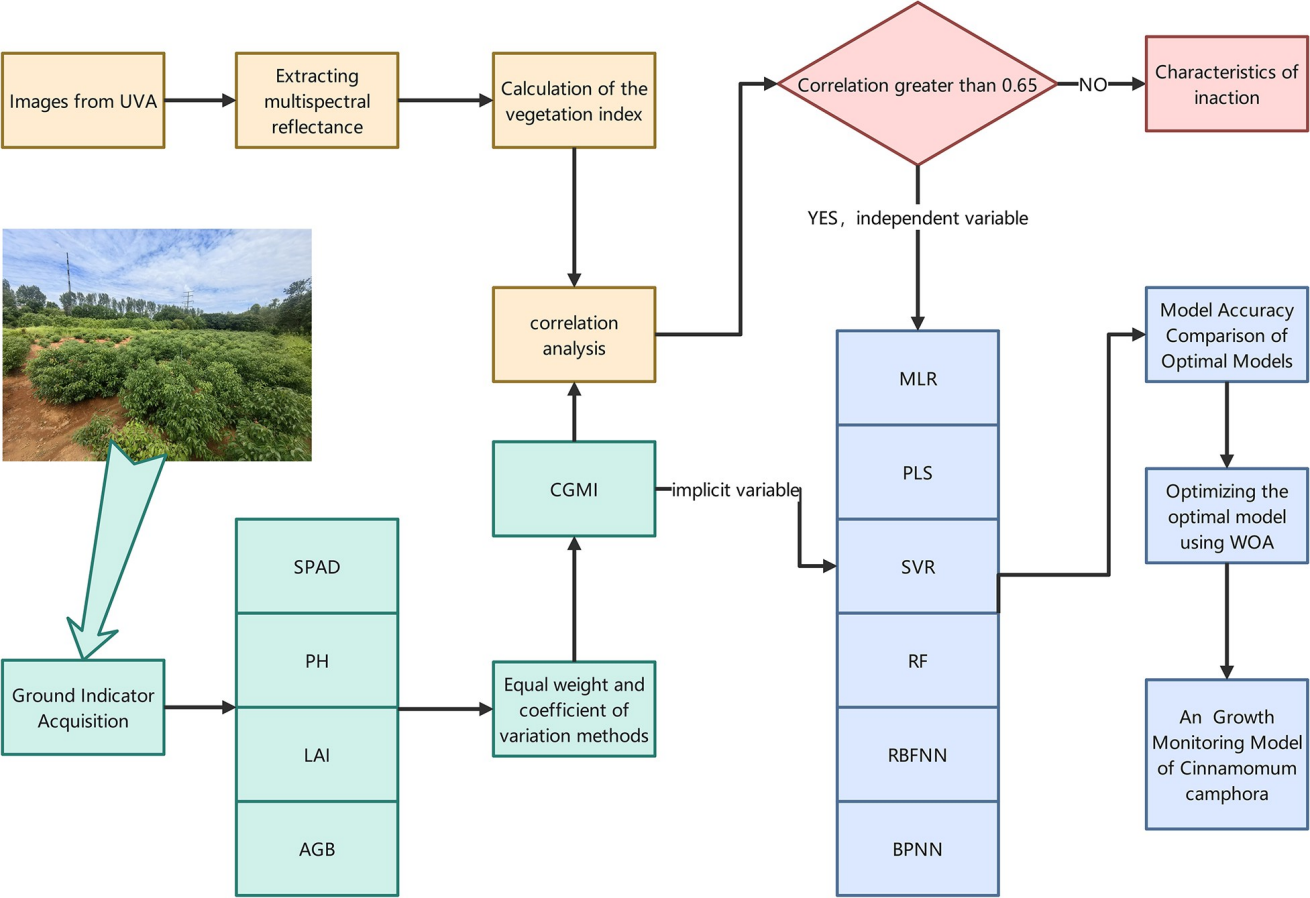
Research framework diagram.

The research framework is shown in the Fig 2 below.

## 3. Results and analysis

### 3.1 Data analysis

According to the CV method, the four single index weights of SPAD, PH, LAI, and AGB were 0.130514, 0.236403, 0.302251, and 0.330832, respectively; therefore, based on Eq (6), the CGMI was:

$${CGMI}_{1}=0.25\;(U_{1}+U_{2}+U_{3}+U_{4})$$ (17)

$${CGMI}_{2}=0.130514U_{1}+0.236403U_{2}+0.302251U_{3}+0.330832U_{4}$$ (18)

where *U*_1_ is the normalized SPAD value, *U*_2_ is the normalized PH, *U*_3_ is the normalized biomass, and *U*_4_ is the normalized plant water content. The statistical results of *C*. *camphora* growth data are shown in Tables 2 and 3. The sample size was 66, and the coefficients of variation of SPAD, PH, LAI, and AGB were 0.09, 0.17, 0.22, and 0.23, respectively. Except for SPAD, the coefficients of variation of single-growth indices were greater than 0.15, indicating that the growth data were significantly affected by the treatments. The coefficients of variation of comprehensive growth indices were over 0.30, at least 0.10 higher than that of single-growth indices, with greater dispersion, which had less impact on the modeling analysis for the algorithm with large noise tolerance.

**Table 3:** Sample statistics.

Name	Minimum value	Maximum value	Average value	Standard deviation	Coefficient of variation
SPAD	33.50	54.78	40.18	3.75	0.09
PH	0.58	1.57	1.18	0.20	0.17
LAI	1.25	4.28	2.90	0.63	0.22
AGB	8720.09	2095.28	6347.70	1515.69	0.23
CGMI_1_	0.06	0.90	0.52	0.16	0.32
CGMI_2_	0.06	0.93	0.55	0.18	0.33

### 3.2 Correlation analysis of vegetation index and growth index

**Fig 3:**
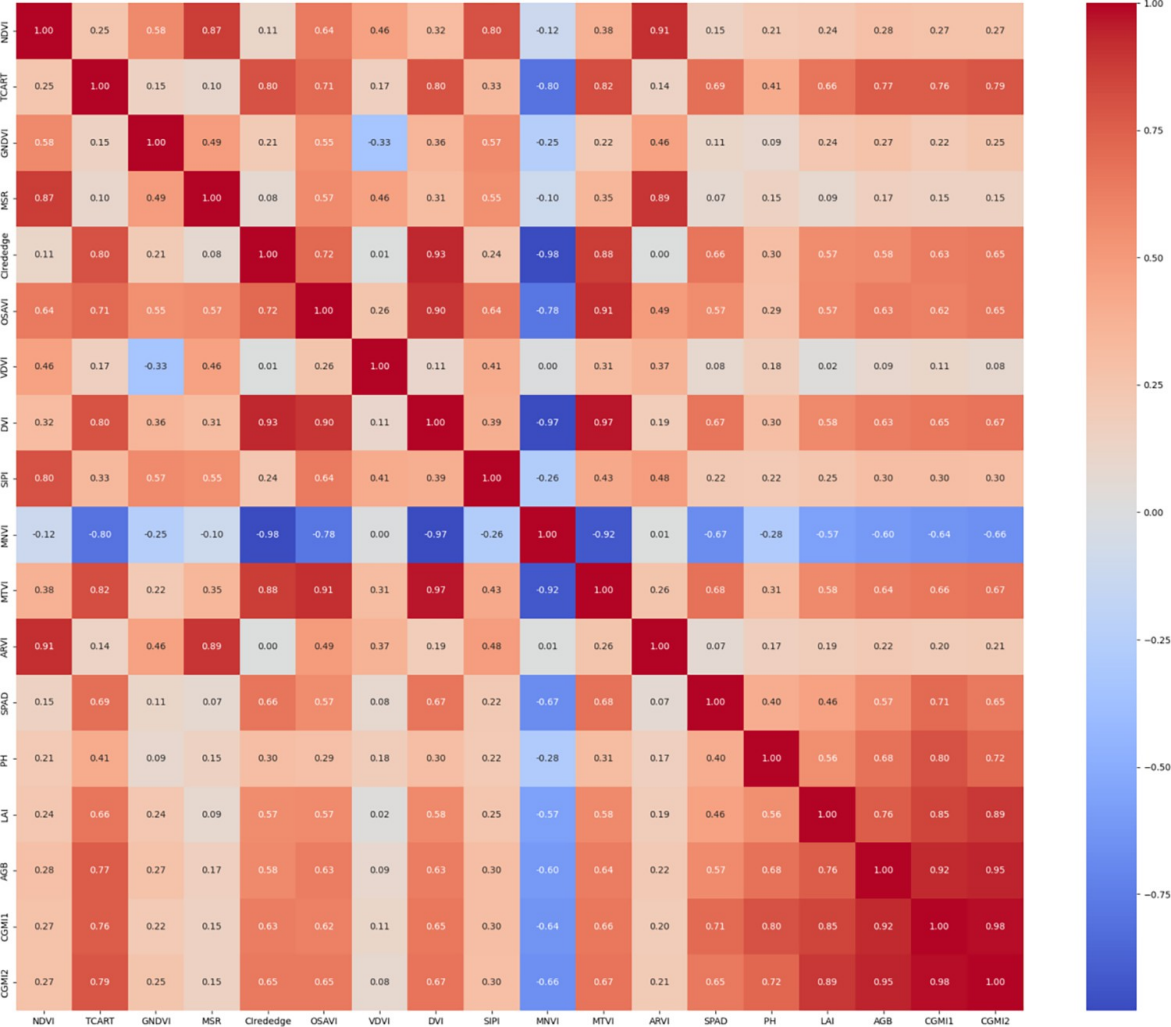
Correlation analysis.

The Pearson correlation coefficients of vegetation index and *C*. *camphora* growth index calculated based on UAV multispectral reflectance are shown in Fig 3, and the correlation coefficients take values in the range of [–1,1]. The correlation coefficients were positively correlated when the two variables increase simultaneously.

It can be seen that some vegetation indices are highly correlated with each other, so in order to reduce the information sink and covariance, only five vegetation indices with high correlation with *C*. *camphora* and significant at 0.01 level, namely, TCART, Cl_rededge_, OSAVI, DVI and MNVI, are selected as model inputs in this study. Among them, TCART is the vegetation index with the highest correlation with *C*. *camphora* ’s growth index, and its correlation with four single growth indexes of SPAD, PH, LAI and AGB, as well as two composite growth indexes of CGMI_1_ and CGMI_2_ are 0.69, 0.41, 0.66, 0.77, 0.76, and 0.79, respectively. compared with the single growth indexes, the correlation of CGMI with the five vegetation indexes which have passed the significance Compared with the single growth index, the correlation between CGMI and the five vegetation indices that passed the significance test was significantly higher, and the improvement of CGMI_2_ was more significant than that of CGMI_1_. The correlation between CGMI_2_ and DVI was 123.33% higher than that between PH and DVI, and the correlation between CGMI_2_ and TCART was 92.45% higher than that between PH and TCART, indicating that the selected vegetation indices could better respond to CGMI_2_.

### 3.3 Inversion model construction

**Fig 4:**
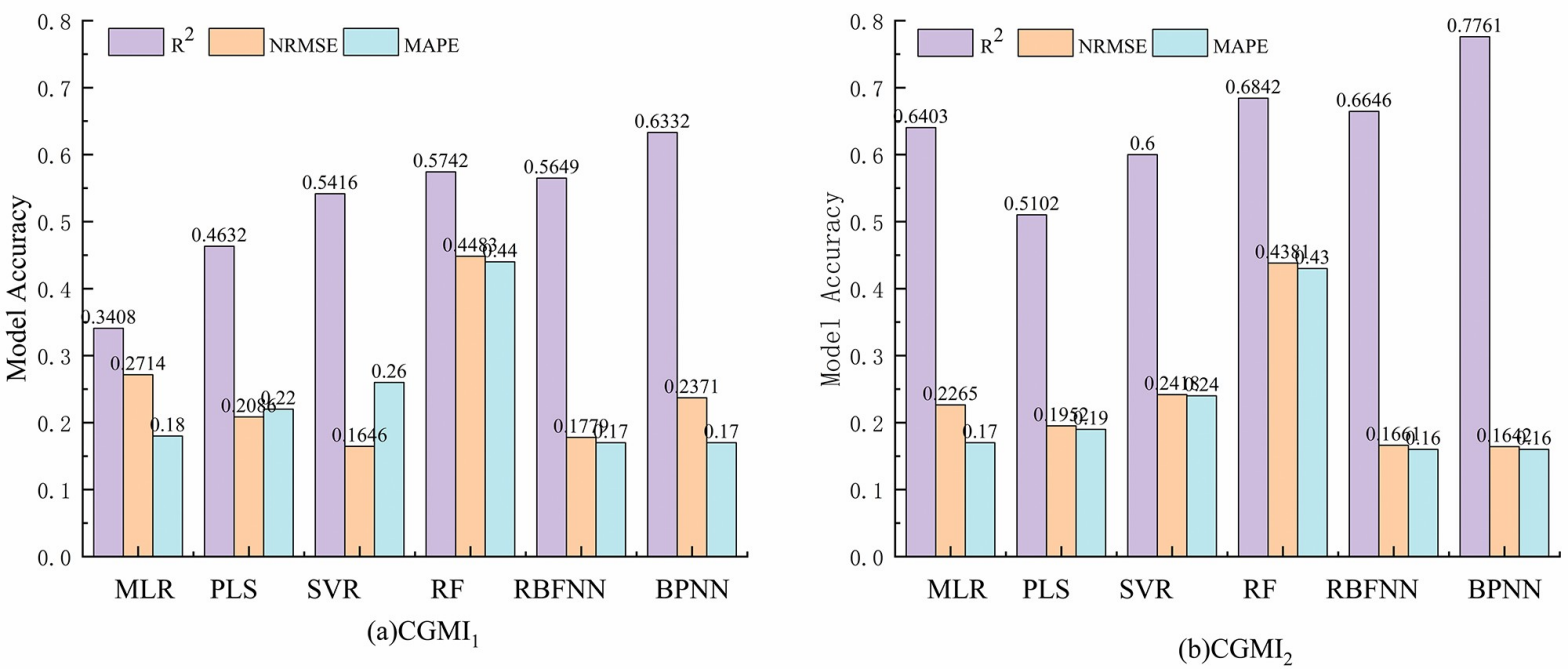
Model evaluation indicators. (a) CGMI_1_ model accuracy; (b) CGMI_2_ model accuracy.

**Fig 5:**
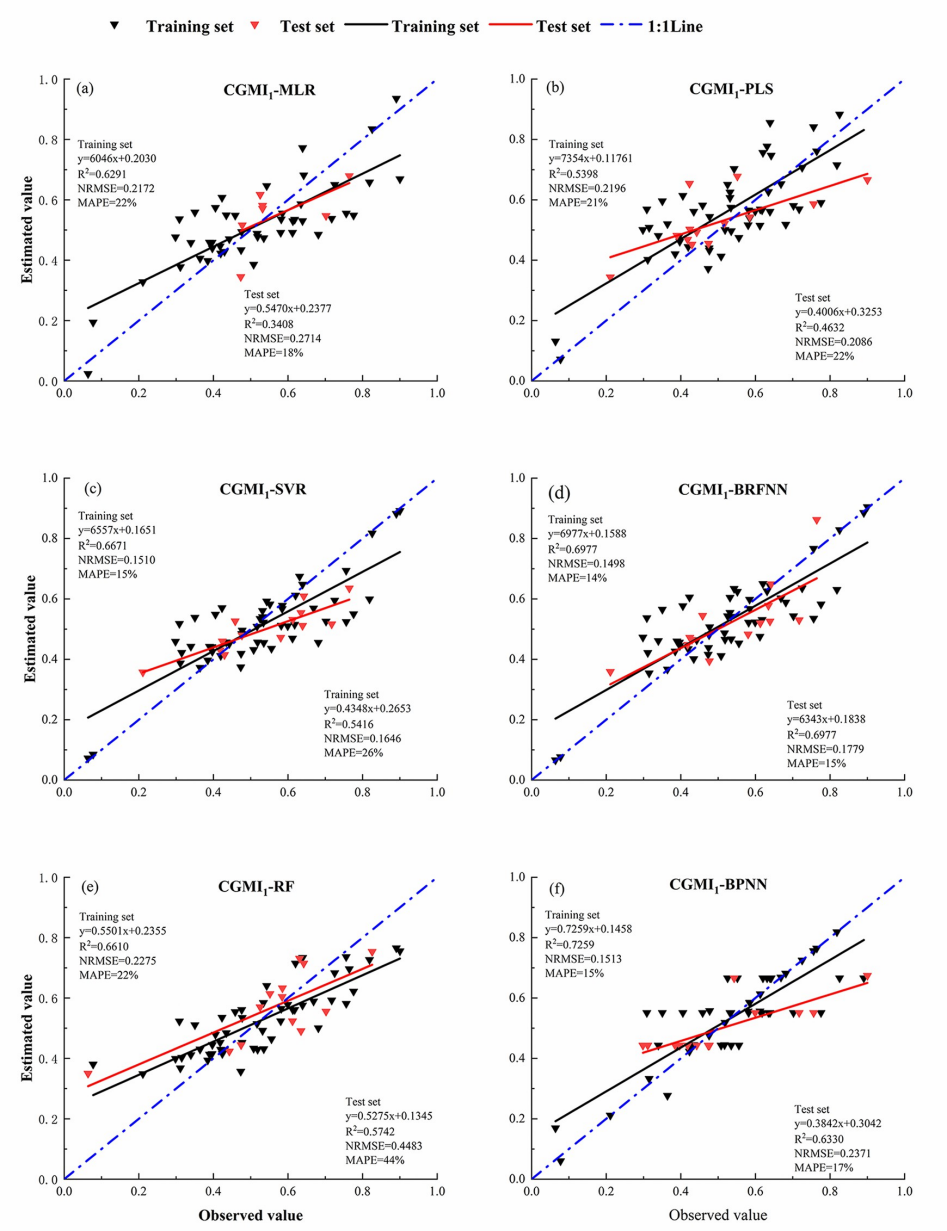
Inversion CGMI_1_ model fitting results. (a) CGMI1 is inverted based on MLR model; (b) CGMI_1_ is inverted based on PLS model; (c) CGMI_1_ is inverted based on SVR model; (d) CGMI_1_ is inverted based on BRFNN model; (e) CGMI_1_ is inverted based on RF model; (f) CGMI_1_ is inverted based on BPNN model.

**Fig 6:**
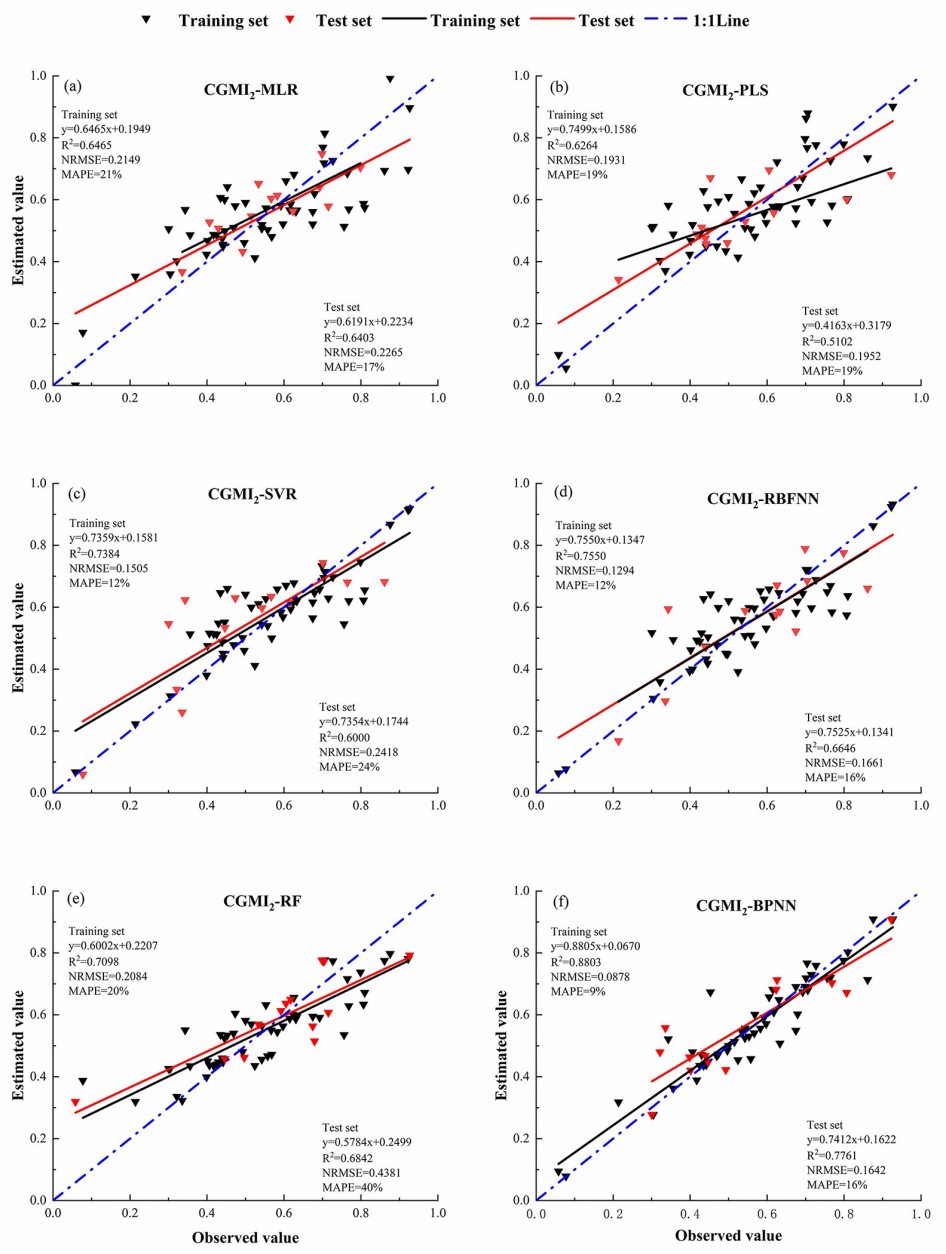
Inversion CGMI_2_ model fitting results. (a) CGMI_2_ is inverted based on MLR model; (b) CGMI_2_ is inverted based on PLS model; (c) CGMI_2_ is inverted based on SVR model; (d) CGMI_2_ is inverted based on RF model; (e) CGMI_2_ is inverted based on BRFNN model; (f) CGMI_2_ is inverted based on BPNN model.

Fifty-two samples were selected as the training set, and 14 as the validation set to estimate CGMI_1_ and CGMI_2_ using six models: MLR, PLS, SVR, RF, RBFNN, and BPNN, and The model accuracy is shown below(Table 4 and Fig 4) and the model fitting results are shown below (Figs 5 and 6):

**Table 4:** Model accuracy evaluation.

	Model Type	*R* ^*2*^		NRMSE		MAPE	
		Training set	Test set	Training set	Test set	Training set	Test set
CGMI_1_	MLR	0.6291	0.3408	0.2172	0.2714	22%	18%^
	PLS	0.5398	0.4632	0.2196	0.2086	21%	22%
	SVR	0.6671	0.5416	0.1510	0.1646	15%	26%
	RF	0.6610	0.5742	0.2275	0.4483	22%	44%
	RBFNN	0.6977	0.5649	0.1498	0.1779	14%	17%
	BPNN	0.7259	0.***6332***	0.1513	0.2371	15%	17%
CGMI_2_	MLR	0.6465	0.6403	0.2149	0.2265	21%	17%
	PLS	0.6264	0.5102	0.1931	0.1952	19%	19%
	SVR	0.7384	0.6000	0.1505	0.2418	12%	24%
	RF	0.7098	0.6842	0.2084	0.4381	20%	43%
	RBFNN	0.7550	0.6646	0.1292	0.1661	12%	16%
	BPNN	0.8803	0.7761	0.0878	0.1642	9%	16%

In the monitoring model of comprehensive growth index of *C*. *camphora* constructed by equal weight method, only the *R*^*2*^ of training set and validation set of BPNN model were greater than 0.6, which were 0.7259 and 0.6332, respectively, NRMSE 0.1513 and 0.2371, and MAPE 15% and 17%, respectively. The second is RBFNN, RF and SVR models, *R*^*2*^ of training set and validation set is both greater than 0.5. And the RBFNN model has low NRMSE and MAPE, with NRMSE of 0.1498 and 0.1779 for the training and validation sets, and MAPE of 14% and 17%, respectively. However, the *R*^*2*^ of MLR and PLS model validation set was lower than 0.5, and the NRMSE was higher, which could not effectively invert the comprehensive growth of *C*. *camphora* dwarf forest.

Among the comprehensive growth index monitoring models constructed using the CV method, the BPNN model exhibited the best accuracy, with the highest training and validation set *R*^*2*^ of 0.8803 and 0.7761, respectively, NRMSE and MAPE are the lowest, with NRMSE of 0.0878 and 0.1642 for the training and validation sets, respectively, and MAPE of 9%h and 16%. respectively. The RF and RBFNN models displayed the next-best accuracy, with training set *R*^*2*^ of 0.7098 and 0.7550, respectively, and validation set *R*^*2*^ of 0.6842 and 0.6646 respectively. The NRMSE and MAPE of the validation sets of the RBFNN model were lower (0.1661 and 16%, respectively), whereas those of the RF model were higher (0.4381 and 0.43%, respectively). The accuracy of the MLR and SVR models were similar, with both the training and validation set *R*^*2*^ being >0.6. Only the PLS model validation set *R*^*2*^ was <0.6, with its training and validation set *R*^*2*^ being 0.6264 and 0.5102, respectively, NRMSE being 0.1931 and 0.1952, respectively, and MAPE of 19% and 19%,respectively.

The comprehensive growth index monitoring model’s accuracy was significantly higher when constructed using the CV method than the equal weighting method, with the MLR model’s accuracy showing the most significant improvement. The validation set *R*^*2*^ and NRMSE of the MLR model constructed using CGMI_2_ improved by 87% and decreased by 16%, respectively, compared with that constructed using CGMI_1_. Following that, compared with the BPNN model constructed using CGMI_1_, that constructed using CGMI_2_ increased both the training and validation set *R*^*2*^ by 21%, decreased NRMSE by 41% and 12%, respectively, and MAPE was reduced by 6% and 1%, respectively. The PLS model exhibited the least change; compared with the PLS model constructed using CGMI_1_, the PLS model constructed using CGMI_2_ increased the training and validation set *R*^*2*^ by 16% and 10%respectively, decreased NRMSE by 12% and 6%, respectively, and MAPE was reduced by 2% and 3%.

### 3.4 Optimizing BPNN

**Fig 7:**
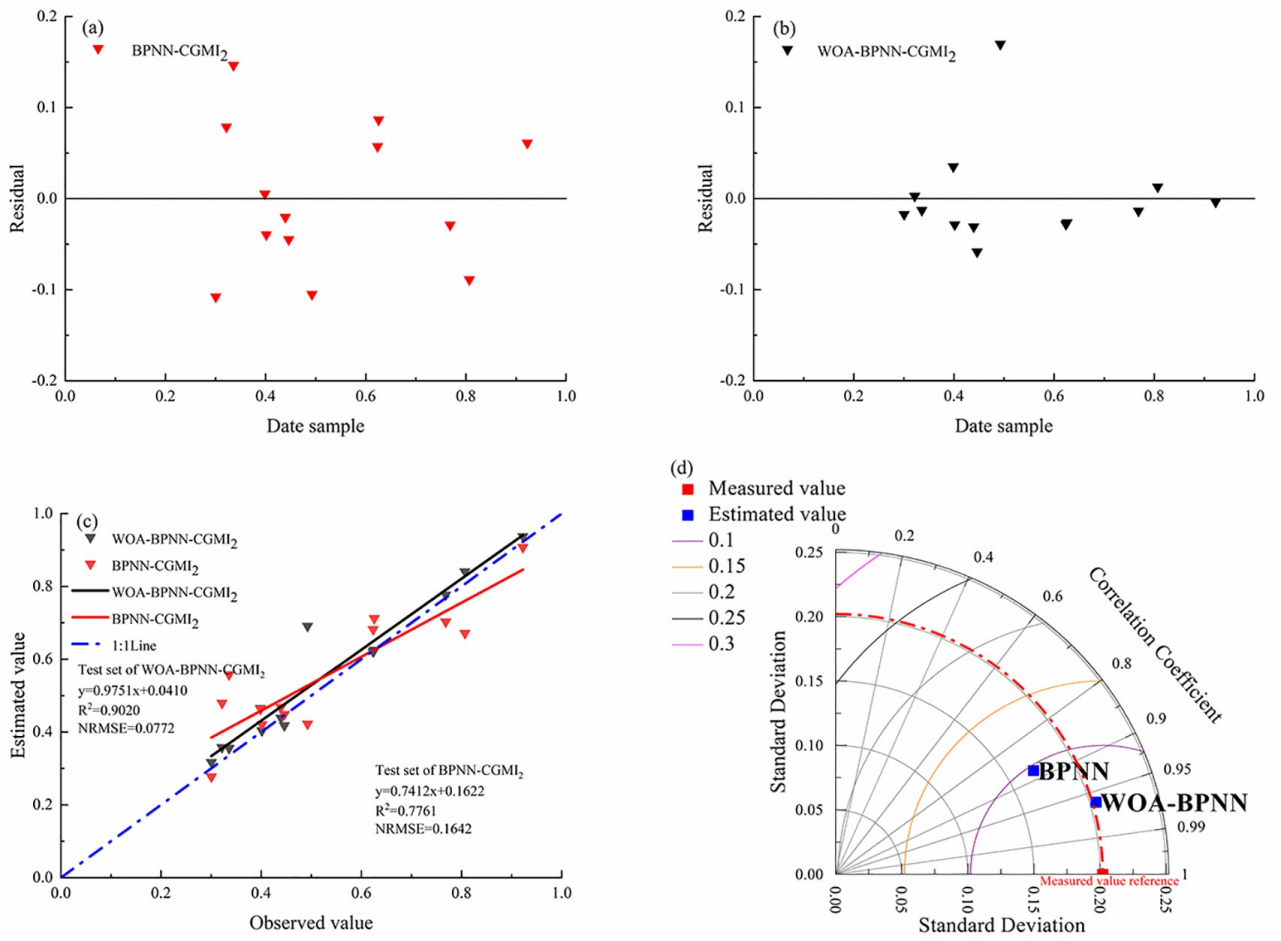
Optimized model fitting results. (a) Residual plot of the BPNN-CGMI_2_; (b) Residual plot of the WOA- BPNN-CGMI_2_; (c) Comparison of the fitting results of the validation set;(d) Taylor diagram of the test set.

The inversion model of *C*. *camphora* growth constructed by combining BPNN with the CV method had high prediction accuracy and satisfactory modeling effect. However, because the training process in deploying the BPNN architecture is challenging owing to the long training time required and low efficiency, this study used the WOA algorithm to optimize the weights and thresholds of the BPNN model while maintaining the remaining BPNN model’s parameter settings intact. The optimization parameters included population size, number of evolutions, number of neurons in the input layer, number of neurons in the output layer, weights from the input layer to the hidden layer, and weights from the hidden layer to the output layer. The initial population size of the WOA-optimized BPNN model was 10, the number of iterations was 60, and the optimized model fitting results are shown in Fig 7.

In the residual plot, it can be observed that the relationship between the residuals of the WOA-BPNN model and the sample data shows a more uniform distribution than the BPNN model. Therefore, according to the performance of the residual plot, it can be concluded that the WOA-BPNN model has better performance than the BPNN model.

The accuracy of WOA-BPNN model optimized by WOA algorithm is significantly higher than that of BPNN model. The *R*^*2*^ of training set and validation set of camphor growth inversion model constructed by WOA-BPNN combined with coefficient of variation method are 0.9960 and 0.9020, respectively, NRMSE are 0.0124 and 0.0722, respectively, and MAPE are 1% and 7%, respectively. The WOA-BPNN model is 13% and 18% higher than the BPNN model training set and validation set *R*^*2*^, respectively, the NRMSE is reduced by 85% and 33%, respectively, and the MAPE is reduced by 7% and 9%. This shows that the WOA algorithm can be used to optimize the growth inversion model of *C*. *camphora* dwarf forest constructed by BPNN and coefficient of variation method. This algorithm can be applied to the establishment of comprehensive growth monitoring model of *C*. *camphora* dwarf forest.

## 4. Discussion

The correlation between the growth and multispectral vegetation indices of *C*. *camphora* revealed that among the four single-growth indices, SPAD, AGB, and LAI had high and similar correlation with the multispectral index, whereas PH had a low correlation with the multispectral vegetation index. The multispectral vegetation index can reflect information on plant chlorophyll content, cover, and density, although PH had no direct relationship with these data; therefore, PH had a low correlation with the multispectral vegetation indices [48]. CGMI_1_ and CGMI_2_ comprehensive growth indices were more correlated with multispectral vegetation indices than the four single-growth indices, presumably because comprehensive growth indices provide more information than single-growth indices. The five vegetation indices: TCART, Cl_rededge_, OSAVI, DVI, and MNVI, were more correlated with *C*. *camphora* growth, with a correlation with CGMI_2_ of 0.79, 0.65, 0.65, 0.67, and 0.67, respectively, indicating that CGMI_2_ correlated more significantly with NIR band multispectral reflectance and chlorophyll-related vegetation indices.

The *C*. *camphora* comprehensive growth monitoring model’s accuracy based on the comprehensive growth index CGMI_2_ of the CV method was significantly higher than that of the model based on the comprehensive growth index CGMI_1_ of the equal weighting method. This result was similar to that of a previous study using the CV method to model the comprehensive growth of winter wheat [9]. This may be because the CV method assigns higher weights to a single index with larger coefficients of variation, whereas the equal weight method assigns the same weight to every vegetation index, making the information in CGMI_2_ more distinguishable and stable than that in CGMI_1_, leading to higher model accuracy built using CGMI_2_.

The BPNN model’s accuracy in the vegetation index was higher than that of the MLR, PLS, SVR, RF, and RBFNN models, indicating that the BPNN model more effectively estimates *C*. *camphora* CGMI than other models. The MLR and PLS models are regression models based on linear assumptions. Although these methods can control the relationship between multiple independent variables and one dependent variable, they cannot adequately capture the nonlinear relationship, resulting in underfitting or overfitting. However, in the case of a nonlinear relationship between vegetation index and comprehensive growth of *C*. *camphora*, the accuracy of the MLR and PLS models may be affected [49, 50]. RF could not solve regression problems as adequately as classification problems and failed to provide a continuous output. When regression analysis is performed, some specific noisy data are prone to overfitting in modeling [51]. SVR and RBFNN models can control nonlinear problems better and have faster training speeds than the BPNN model; however, they are mainly applicable to low-dimensional datasets, and their training time and computational cost are higher for high-dimensional datasets, which may affect the accuracy of SVR and RBFNN models [51, 52]. The higher BPNN model accuracy may be attributed to its high nonlinear function approximation capability, self-learning and self-adaptive capabilities, and fault tolerance to cope with measurement errors, making it more suitable for the inversion of CGMI of *C*. *camphora* than other models [53].

The integrated growth model based on WOA optimization improved *R*^*2*^ by 12% compared with the previous model for SPAD inversion only [21], indicating that CGMI based on WOA optimization is more advantageous than a single growth index for dwarf forest arborvitae growth inversion. After replacing the BPNN randomly assigned weights and thresholds with the optimal weights and thresholds obtained by the WOA algorithm while keeping the remaining model parameters consistent, the model *R*^*2*^ and stability were significantly improved, and NRMSE was significantly decreased. Furthermore, the estimated values of the WOA–BPNN model were closer to the measured values compared with those of the BPNN model after plotting scatter plots with the same data, and the model accuracy was improved, indicating that the WOA algorithm can improve the prediction accuracy of the CGMI2–BPNN model for *C*. *camphora*.

The inversion model derived from this study only applies to the present measurement data, and further research is needed to estimate the CGMI of *C*. *camphora* in different growth cycles, species, regions, and periods. In addition, the approximation of calculation formulae and some spectral information, which may produce numerous highly correlated vegetation indices when six narrowband multispectral sensors are used, may cause information redundancy and co-linear effects in the model. This study attempted to avoid this problem through vegetation indices screening. Therefore, future model optimization studies can increase the sample size and use hyperspectral technology to screen the spectral bands and vegetation indices that are more sensitive to the *C*. *camphora* growth index. Furthermore, we discussed and developed models for different *C*. *camphora* species and growth periods to obtain a more accurate and comprehensive *C*. *camphora* growth inversion model.

## 5. Conclusions

In this study, we constructed CGMI based on field and laboratory data, analyzed the correlation between CGMI and selected vegetation indices using the basic principles of the CV method and traditional weighting method, and combined the constructed CGMI_1,_ CGMI2, and UAV multispectral data with various growth analysis models and WOA model to achieve effective monitoring of *C*. *camphora* growth in the study area. Specific conclusions were obtained as follows:

The correlation analysis of the obtained data of SPAD, PH, LAI, and AGB of *C*. *camphora*, combined with different weighting methods, showed that the correlation of the constructed CGMI increased to different degrees compared with single-growth indices, and most of them showed significant correlation. The TCART, Cl_rededge_, OSAVI, DVI, and MNVI were highly correlated with *C*. *camphora* growth indices, their correlations with CGMI2 were 0.79, 0.65, 0.65, 0.67 and 0.67, respectively.

The CV method could better reverse the comprehensive growth of *C*. *camphora* than the equal weight method. In the model of comprehensive growth indicators of *C*. *camphora* constructed by the equal weight method, the validation set *R*^*2*^ of MLR, PLS, SVR, RF, RBFNN and BPNN models were 0.3408, 0.4632, 0.5416, 0.5742, 0.5649, and 0.6332, respectively; and in the model of comprehensive growth indicators of arborvitae constructed by combining with the method of coefficient of variation The coefficients of determination *R*^*2*^ were 0.6403, 0.5102, 0.6000, 0.6840, 0.6646, and 0.7761, respectively.

The training set and validation set *R*^*2*^ of the WOA-BPNN model with the coefficient of variation method were 0.9960 and 0.9020, the NRMSE was 0.0124 and 0.0722, respectively, and MEAP are 1% and 7%, respectively. The WOA–BPNN model’s accuracy optimized using the WOA algorithm was significantly improved compared with the BPNN model. The coefficient of determination increased by 18% and the NRMSE decreased by 33% compared with the BPNN model. Therefore, the WOA–BPNN model is the best model to invert the growth potential of *C*. *camphora*.

Future modeling studies could increase the sample size and discuss the model for different growth periods of *C*. *camphora* and try new algorithms to optimize the model as well as screening vegetation and texture indices that are more sensitive to camphor pine growth indices.

## Supporting information

S1 Data (XLSX)

S2 Data (XLSX)

S3 Data (XLSX)

S1 File (ZIP)
