# Supplementary figures and images for: An integrated UAV growth monitoring model of Cinnamomum camphora based on whale optimization algorithm
Source: PLoS One. 2024 Jun 21;19(6):e0299362. doi: 10.1371/journal.pone.0299362 (PMC11192338; doi:10.1371/journal.pone.0299362)

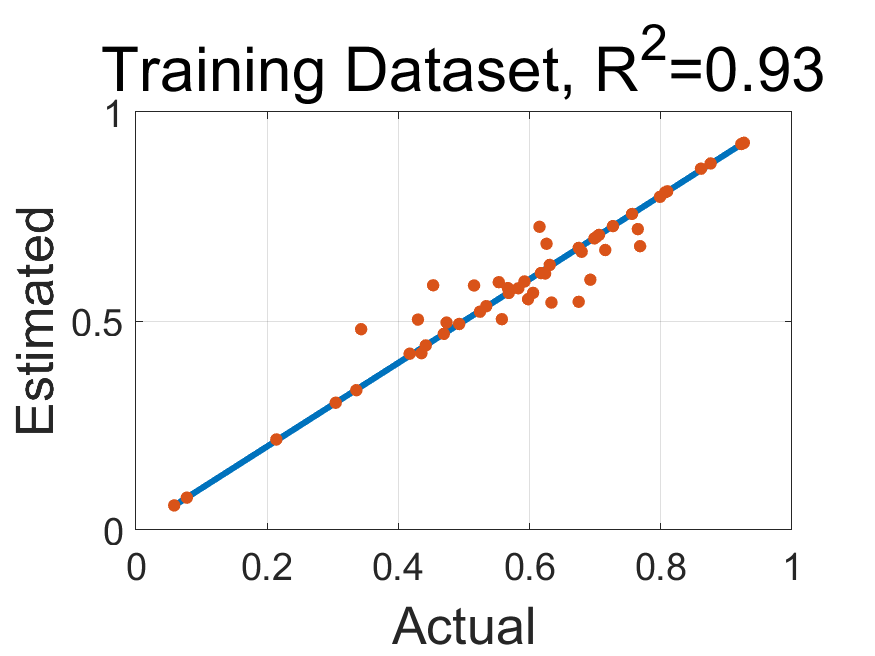

Supplement: S1 File — (ZIP) [file pone.0299362.s004.zip › 程序/PLS/ScatterDiagram.png]
